# Minimum dataset for monitoring national human immunodeficiency virus pre-exposure prophylaxis (HIV PrEP) programmes: a five-nation consensus, 2019

**DOI:** 10.2807/1560-7917.ES.2021.26.23.2001595

**Published:** 2021-06-10

**Authors:** John Saunders, O Noel Gill, Valerie Delpech, Claudia Estcourt, the Five Nations (England, Scotland, Wales, Northern Ireland, Republic of Ireland) PrEP Minimum Dataset Working Group, Five Nations (England, Scotland, Wales, Northern Ireland, Republic of Ireland) PrEP Minimum Dataset Working Group, Five Nations (England, Scotland, Wales, Northern Ireland, Republic of Ireland) PrEP Minimum Dataset Working Group, Ann Sullivan, Dana Ogaz, Alison Brown, Ada Miltz, Adamma Aghaizu, Naresh Chada, Caroline Hurley, Fiona Lyons, Nicola Steedman, David Goldberg, Lesley Wallace, Duncan McMaster, Zoe Couzens, Laia Fina

**Affiliations:** 1 National Infection Service, Public Health England, London, United Kingdom; 2 School of Health and Life Sciences, Glasgow Caledonian University, Cowcaddens, Glasgow, United Kingdom; 3 The members of the Working Group are listed under Investigators

**Keywords:** Human Immunodeficiency Virus, Pre-Exposure Prophylaxis, Surveillance, Epidemiology

## Abstract

Human immunodeficiency virus (HIV) pre-exposure prophylaxis (PrEP), the use of antiretroviral medication to prevent HIV acquisition, is a highly effective biomedical prevention tool. The World Health Organization (WHO) recommends PrEP for people at substantial risk of HIV infection, as part of combination prevention, and highlights the need for robust evaluation of PrEP programmes. Based on suggested WHO core indicators, we created a concise set of HIV PrEP-related dataset variables, to harmonise the monitoring and evaluation of PrEP programmes across five closely related nations (England, Northern Ireland, Ireland, Scotland and Wales). The dataset is based on the PrEP cascade and is intended to represent the minimum variables needed for reporting and comparison of meaningful data at national and multinational level. The dataset can be modified for settings with different health and surveillance systems. It is intended for public health, academic, clinical and health planning, and public audiences. Here we describe the dataset and illustrate its use with data from the first year of the Scottish National PrEP programme.

## Context

Human immunodeficiency virus (HIV) pre-exposure prophylaxis (PrEP), the use of antiretroviral medication to prevent HIV acquisition, is a highly effective biomedical prevention tool [1-3]. The World Health Organization (WHO) recommends PrEP for people at substantial risk of HIV infection as part of evaluated combination prevention approaches to reduce HIV transmission [4].

Some countries have well established programmes whereas others, including many countries with high HIV incidence among key populations, have yet to implement PrEP services [5]. State-funded PrEP services were rolled out between 2017 and 2019 in Northern Ireland, Scotland and Wales. In England, PrEP was initially delivered through a large-scale implementation trial (The PrEP Impact Trial) between 2017 and 2020 [6]. PrEP is delivered through sexual health clinics. Each devolved nation has responsibility for its own publicly funded healthcare system but the model for sexual health services is broadly similar (free, confidential and open to anyone without the need to be resident in the local area, registered with or referred by a primary healthcare physician). Although not part of the UK, Ireland, a close neighbour, has a similar healthcare system and implemented a national PrEP programme in November 2019. The five countries have broadly similar eligibility criteria for state-funded PrEP (differences reflect local epidemiology) (Box) and separate surveillance systems to monitor and evaluate sexual health and PrEP delivery.

Box **PrEP eligibility (prescribing) criteria, United Kingdom and Ireland, 2019**

**Table:** 

**England and Wales [** 13 **]**
Age 16 years or older, HIV-negative, (Wales only: resident in Wales). Plus one or more of the following criteria: Individuals, irrespective of gender, who report condomless sex with HIV-positive partners, unless the partner has been on antiretroviral therapy for at least 6 months and their plasma viral load is < 200 copies/mL, Cis- and transgender gay, bisexual men and other men who have sex with men, and transgender women reporting condomless anal sex in the last 6 months and ongoing condomless anal sex, Individuals, irrespective of gender, clinically assessed as being at an equivalent risk of HIV acquisition as those in the groups above.
**Northern Ireland**
Age 16 years or older, Able to attend regular 3-month reviews in Belfast (or Derry/Londonderry), Willing to stop taking PrEP when no longer eligible, HIV-negative and tested for HIV at a clinic in the previous 12 months, Resident in Northern Ireland. Plus one or more of the following criteria: Current sexual partners, irrespective of gender, of HIV-positive people who have a detectable viral load, Cis- and transgender gay, bisexual men and other men who have sex with men, and transgender women reporting condomless anal sex in the last 3 months and likely to do so again in the next 3 months, Individuals, irrespective of gender, clinically assessed as being at an equivalent highest risk of HIV acquisition.
**Scotland**
Age 16 years or older, A confirmed HIV-negative test in a sexual health clinic, Able to attend regular 3-month reviews, Willing to stop taking PrEP when no longer eligible, Resident in Scotland. Plus one or more of the following criteria: Current sexual partners, irrespective of gender, of HIV-positive people who have a detectable viral load, Cis- and transgender gay and bisexual men, other men who have sex with men, and transgender women with a documented bacterial rectal sexually transmitted infection in the last 12 months, Cis- and transgender gay, bisexual men and other men who have sex with men, and transgender women reporting condomless penetrative anal sex with two or more partners in the last 12 months and likely to do so again in the next 3 months, Individuals, irrespective of gender, at an equivalent highest risk of HIV acquisition, as agreed with another specialist clinician.
**Ireland**
Age 17 years or older, HIV-negative, Resident in Ireland. Plus one or more of the following criteria: Men who have sex with men or transgender women who have sex with men reporting condomless anal sex with at least two partners over the last 6 months, Men who have sex with men or transgender women who have sex with men and have had a documented or reported acute sexually transmitted infection in the last 12 months, Men who have sex with men or transgender women who have sex with men and have documented or reported use of HIV post-exposure prophylaxis following sexual exposure over the last 12 months, Men who have sex with men or transgender women who have sex with men and who reported engagement in chemsex over the last 6 months, Individuals having condomless sex with an HIV-positive person who is not stably suppressed on antiretroviral therapy, Other heterosexual men or heterosexual women considered by a senior clinician specialising in HIV medicine to be at substantial risk for sexual acquisition of HIV.

HIV: human immunodeficiency virus; PrEP: pre-exposure prophylaxis.

Evaluation is a key part of combination HIV prevention [7]. In the absence of agreed national monitoring frameworks, we formulated a pragmatic framework for evaluation which facilitated consistency and comparability of data items across the UK and Ireland, mindful of the different contexts and stages of PrEP implementation. These variables build on suggested core indicators proposed by WHO [8]. The dataset is intended to represent the minimum reporting variables needed for review of meaningful data at national level and harmonised reporting across the UK and Ireland. It is designed to be of use to public health, academic, clinical and health planning, and public audiences. The minimum dataset is not intended to be an exhaustive evaluation of PrEP delivery in each nation. Rather, it uses routine surveillance items to monitor key stages in PrEP delivery within and between each country to inform interventions (e.g. to increase PrEP uptake in key populations that are underrepresented).

Here we describe the dataset and underpinning methods, justify the variables included, and provide an illustrative example of its use with national-level reporting of the first year of the national PrEP programme in Scotland (the first of the UK nations to implement PrEP).

## Process for developing the dataset

**Figure 1:**
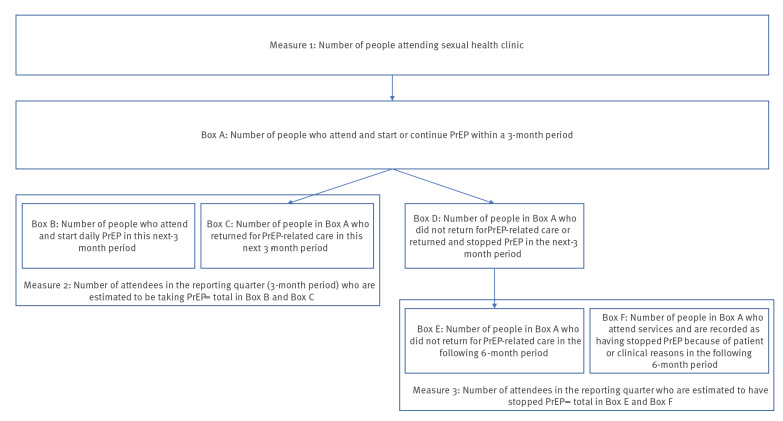
Steps in the oral PrEP cascade (adapted WHO implementation tool for PrEP of HIV infection [8])

HIV: human immunodeficiency virus; PrEP: pre-exposure prophylaxis; WHO: World Health Organization.

We created an initial draft dataset based on the PrEP cascade (analogous to the HIV treatment continuum of care [8]) within the PrEP monitoring and evaluation module of the WHO implementation tool for PrEP of HIV infection, to give structure to the dataset and ensure consideration of data items from all key steps. The six PrEP cascade steps are: (1) identify high-risk potential PrEP candidates, (2) determine eligibility and interest, (3) initiate PrEP, (4) achieve adherence, (5) continue PrEP and (6) stop PrEP (Figure 1). These steps can help to identify, and therefore address, gaps or inequities in uninterrupted PrEP delivery. 

The draft dataset contained a number of data items, derived from routine epidemiological practice, for each stage of the cascade. We adopted a modified Delphi technique of discussion and revision [9] with public health, epidemiology, clinical academic and health planning experts from all five nations (five to 10 participants per round). Over three rounds, we obtained consensus on a final dataset of minimum meaningful data elements which covered all but one stage of the PrEP cascade. Although each step of the cascade was considered, the group could not agree a suitable way to standardise some of these. For example, because prescribing criteria for PrEP differs between nations, a standardised measure to identify potential PrEP candidates and eligibility was not possible. Another example was adherence: although the group considered indirect measures of adherence using surveillance datasets (e.g. the proportion of days covered by a prescription), individuals on event-based dosing, or who are stopping and starting daily PrEP appropriately according to periods of risk, would be incorrectly identified as non-adherent. Therefore, it was decided to focus on continuation. 

The consequence of this is that direct comparison of need, and more importantly, unmet need, will be challenging between countries. However, the minimum dataset is not intended to describe the total monitoring and evaluation efforts for each nation and countries will be assessing likely PrEP need among their residents to inform delivery.

Ethical approval was not required for developing these proposed variables.

## Proposed minimum dataset

Four key variables and disaggregated measures are included with data to be aggregated into reporting quarters (RQ) and combined for annual reporting. These variables map broadly to steps 1, 3, 4, 5 and 6 of the PrEP cascade (Figure 1). Step 2, ‘Determine eligibility and interest’, could not be included in our proposed minimum dataset owing to the varying approaches used to determine this across the UK and Ireland. During rounds of discussion, participants noted that, in practice, individual countries and territories will supplement the other five cascade measures with additional information about PrEP users, relevant to country-specific requirements for PrEP surveillance reporting. For example, using country-specific prescribing criteria to estimate potential PrEP candidacy among key populations, national audits and periodical cross-sectional surveys to assess interest in PrEP and adherence.

There were challenges with creating this minimum dataset that needed to be addressed. For example, the proposed measures do not map comprehensively with the PrEP cascade. Measurement of population need is challenging and is likely to require additional data sources to supplement routine surveillance. Accepted definitions do not yet exist to enable consistent measurement of important factors such as PrEP discontinuation or seroconversion of people taking PrEP. As part of developing the dataset we created usable definitions for our nations, but more work will be needed to refine them.

### Measure 1: Number of people attending sexual health clinic

In all the UK nations and Ireland, PrEP is delivered through existing specialist sexual health services. These are state-funded clinics which provide free sexual and reproductive healthcare which includes care for sexually transmitted infection, reproductive healthcare and contraception, and variably HIV outpatient care and other services such as psychosexual care, care after sexual assault and gender services. To inform the denominators for subsequent variables, an understanding of attendance at these services providing PrEP is required, disaggregated by key socio-demographic variables. This measure is not perfect as it is not entirely analogous with PrEP need. However, attendees at sexual health services in our nations are known to be at higher risk of sexually transmitted infections and HIV compared with the general population [10].

This measure has the advantage that it is easily calculated and, therefore, easily standardised across the nations and reported. The main disadvantage is that any fluctuations in numbers of attendees may not indicate a true change in PrEP need. Individual nations will need to create measures for need based on their individual prescribing criteria and knowledge of their country-specific epidemics.

### Measure 2: Number of attendees in the reporting quarter who are estimated to be taking PrEP

Related, but not directly comparable, to steps 3, 4 and 5 in the cascade, the number of clinic attendees receiving a PrEP prescription or attending for PrEP-related care (for clinic-prescribed or privately sourced PrEP) is calculated. The measure gives an indication of the numbers of people using PrEP, which will include a mix of new starters, re-starters and continuations. The numerator is an attempt to capture PrEP users who continue to take PrEP but are not attending exactly every 3 months as per clinical guidance. For the purposes of the harmonised dataset, this is reported by calendar quarter and disaggregated by key sociodemographic variables.

**Figure 2:**
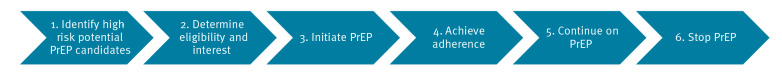
Visual representation for calculating proposed measures of PrEP programmes

The numerator is generated by counting the number of people who attend services and are recorded as having received a PrEP prescription or sourced PrEP privately PLUS any separate additional attendees in the quarter immediately prior (i.e. RQ minus 1) who received a PrEP prescription or who attended for PrEP care (i.e. PrEP sourced privately and clinic attended for monitoring) (Figure 2). A suggested denominator for this measure is the number of people attending services in that quarter (Measure 1). 

PrEP: pre-exposure prophylaxis.

We are likely to underestimate the number of people on PrEP within this step because a proportion of individuals will self-source PrEP from the Internet or from private practitioners. Some of these people privately sourcing PrEP will not be attending publicly funded services for any PrEP-related care. However, we anticipate that individuals are likely to switch from private to publicly funded PrEP as access improves over time. In addition, some supplementary data exist from community-based surveys to help estimate the proportion of PrEP users who source this privately [11].

### Measure 3: Number of attendees in the reporting quarter who are estimated to have stopped PrEP

Some measure of PrEP continuation is important in monitoring and evaluating PrEP programmes and each of the five nations have implemented surveillance codes for people stopping PrEP. In addition to this, for the minimum dataset, we attempt to estimate the number of PrEP users with insufficient PrEP to cover the reporting quarters of interest by assuming that those prescribed 3 months of PrEP must attend in the following 6 months or have stopped. In this way, we also take into account possible switching from daily to event-based PrEP. In the future, it will be possible to compare the numbers in the proposed indicator with the numbers coded as stopped in surveillance datasets to gain an impression of the comparability of these items.

The numerator is generated by counting the number of people who attend services and are recorded as having stopped PrEP PLUS the number who began or renewed daily PrEP in RQ minus 2 and have not returned in the current RQ and RQ minus 1 (i.e. in the 6 months following a PrEP prescription) PLUS the number who began or renewed event-based dosing PrEP in RQ minus 3 and have not returned in the current RQ and RQ minus 1 and RQ minus 2. A suggested denominator for this measure is the estimated number of people attending services in the previous quarter who are on PrEP (Measure 2). 

We are unable to rely solely on clinical coding within surveillance data to accurately quantify PrEP discontinuations, so additional measures (calculating the number who have not returned within 6 months of a prescription for daily PrEP) were incorporated to estimate this. However, these measures are likely to overestimate PrEP discontinuations and interruptions because they assume that individuals who may switch from daily to event-based PrEP dosing (without returning to clinic) are no longer taking PrEP. In addition, within any defined engagement period, individuals may stop PrEP but later restart PrEP or switch between daily and event-based dosing. Longer periods of engagement increase the opportunity for these changes in PrEP use and the potential for them to impact on the interpretation of discontinuation rates.

### Measure 4: Number of new HIV diagnoses and observed recent seroconversions

As PrEP implementation scales up across the five nations, it is anticipated that the number of new HIV infections and diagnoses will continue to decline. The case definitions for ‘HIV diagnoses and seroconversions in PrEP users’ are yet to be standardised and are the subject of active discussion internationally. The WHO defines HIV positivity among people who have been prescribed PrEP as the “*percentage of people who test HIV-positive among people who received PrEP at least once in the last 12 months and had at least one follow-up HIV test”* [8]. Each of the five nations will aim to report new and recent seroconversions in total as well as among those on PrEP per quarter, acknowledging that any true PrEP breakthrough infection will be a small minority of these. Recent seroconversions are defined as those in individuals with a negative HIV test in the previous 12 months and/or evidence of recent HIV infection using a recent infection testing algorithm [12]. The number of new diagnoses can be reported per 10,000 population. 

The accuracy with which PrEP users who acquire HIV can be identified within the nations’ different surveillance systems varies because not all have unique patient identifiers across both sexual health and HIV datasets. Therefore, it is possible that new HIV diagnoses among PrEP users will not be identified for this measure. However, for nations where this is the case, probabilistic linkage of sexual health and HIV datasets has been used and can reduce the risk of missing breakthrough infections.

## The minimum dataset in practice

The Table illustrates use of the minimum dataset across the proposed measures with data from Scotland’s first year of PrEP implementation. These data are used on a rolling basis to inform further development of the programme and service delivery.

**Table:** The PrEP minimum dataset across the proposed measures with data from Scotland’s first year of PrEP implementation, 1 July 2017–30 June 2018 (n =1,874 individuals attending for PrEP)

	1. Sexual health clinic attendees				2. Number using PrEP				3. Number of attendees stopping PrEP				4. Number of new HIV diagnoses and (observed recent seroconversions)			
Calendar quarter	Q1	Q2	Q3	Q4	Q1	Q2	Q3	Q4	Q1	Q2	Q3	Q4	Q1	Q2	Q3	Q4
Scotland	53,293	52,575	53,609	54,118	397	1,019	1,460	1,658	^a^	^a^	65	181	60 (11)	51 (9)	48 (10)	40 (7)
Region of attendance																
Ayrshire and Arran	4,180	4,149	4,319	4,323	9	25	39	45	0	0	^a^	^a^	^a^	^a^	^a^	^a^
Borders	924	893	891	996	^a^	^a^	7	10	0	0	^a^	^a^	^a^	^a^	^a^	^a^
Dumfries and Galloway	1,093	1,025	1,018	1,151	^a^	^a^	11	12	^a^	^a^	0	^a^	^a^	^a^	^a^	^a^
Fife	4,167	3,888	3,840	4,060	10	40	51	50	0	^a^	^a^	13	^a^	^a^	^a^	^a^
Forth Valley	1,956	1,678	1,717	1,842	16	28	40	47	^a^	^a^	^a^	^a^	^a^	^a^	^a^	^a^
Grampian	3,380	3,417	3,663	3,544	39	80	98	121	0	0	8	22	^a^	^a^	^a^	^a^
Greater Glasgow and Clyde	17,272	17,277	17,428	17,227	173	460	674	779	0	7	26	73	^a^	^a^	^a^	^a^
Highland	1,610	1,532	1,675	1,678	8	18	31	34	0	0	^a^	^a^	^a^	^a^	^a^	^a^
Lanarkshire	5,409	5,184	5,253	5,266	12	30	41	41	0	0	^a^	^a^	^a^	^a^	^a^	^a^
Lothian	9,815	10,205	10,315	10,597	95	274	407	446	^a^	^a^	11	43	^a^	^a^	^a^	^a^
Tayside	3,673	3,475	3,636	3,601	27	53	67	77	^a^	^a^	6	9	^a^	^a^	^a^	^a^
Sexual orientation, gender and ethnicity																
Men who have sex with men	4,937	5,281	5,530	5,721	323	1,009	1,442	1,637	^a^	^a^	63	178	27 (5)	23 (7)	21 (^a^)	21 (^a^)
Heterosexual women	26,210	25,182	25,707	25,590	^a^	5	7	7	^a^	0	0	^a^	14 (^a^)	13 (^a^)	12 (^a^)	8 (0)
Black African heterosexual women	173	166	177	167	^a^	^a^	^a^	^a^	^a^	^a^	^a^	^a^	8 (^a^)	6 (^a^)	6 (0)	^a^ (0)
Heterosexual men	7,674	7,310	7,176	7,627	^a^	^a^	5	5	^a^	^a^	^a^	0	7 (^a^)	11 (0)	10 (^a^)	7 (0)
Black African heterosexual men	94	101	96	103	0	0	0	0	0	0	0	0	0 (0)	^a^ (0)	6 (^a^)	^a^ (0)
Transgender and diverse gender	45	41	63	67	^a^	^a^	^a^	6	0	0	0	0	^a ^	^a^	^a^	^a^
Age group (years)																
15–24	19,924	20,094	20,743	20,242	71	184	286	326	^a^	^a^	26	48	6 (^a^)	^a^ (^a^)	5 (^a^)	5 (^a^)
25–34	16,697	16,138	16,716	16,700	146	383	559	641	^a^	^a^	18	71	20 (^a^)	20 (^a^)	16 (^a^)	8 (0)
35–39	5,376	5,241	5,206	5,570	57	136	186	204	0	0	8	24	8 (^a^)	^a^ (0)	8 (0)	8 (^a^)
40–44	3,676	3,624	3,583	3,767	35	87	115	138	^a^	^a^	7	14	8 (5)	5 (^a^)	8 (^a^)	3 (^a^)
45–49	3,089	3,088	3,012	3,121	30	78	115	121	0	^a^	^a^	10	5 (^a^)	5 (0)	5 (^a^)	6 (0)
≥ 50	4,184	4,058	4,006	4,339	58	151	199	228	0	^a^	^a^	14	13 (^a^)	16 (^a^)	6 (^a^)	9 (^a^)

HIV: human immunodeficiency virus; PrEP: pre-exposure prophylaxis; Q: calendar quarter.

^a^ Indicates cells where numbers have been suppressed to prevent potential risk of disclosure (values under 5).

An individual can attend multiple region NHS Boards in each quarter but is counted once in the total. Therefore, the sum of each region will not equal the Scotland total. Individuals aged 0–14 years are included in the totals for each quarter but not shown in the disaggregation by age in the table.

## Conclusions

Robust evaluation of PrEP programmes is essential to understanding their effectiveness and place within wider combination prevention strategies. This expert consensus dataset provides a pragmatic means of evaluating core elements of PrEP programmes for ongoing monitoring and inter-country comparison. Additional items may be added in line with local need and their lack of inclusion within this proposed dataset is not meant to indicate their lack of importance. For example, monitoring side effects, toxicity and resistance mutations among PrEP users who acquire HIV are all important and each nation does have mechanisms and processes in place to look at these. Our hope is that the dataset may assist countries with well-established PrEP programmes and those at earlier stages of development to implement systems collecting comparable data. However, we acknowledge the variation in health systems across Europe and that it may not be feasible to collect all the variables in our proposed minimum dataset. All five nations are working towards reporting to this dataset; this will enable robust data comparisons to advance knowledge of HIV prevention outcomes across these countries and territories.

## References

[1] McCormack S Dunn DT Desai M Dolling DI Gafos M Gilson R Pre-exposure prophylaxis to prevent the acquisition of HIV-1 infection (PROUD): effectiveness results from the pilot phase of a pragmatic open-label randomised trial. Lancet. 2016;387(10013):53-60. 10.1016/S0140-6736(15)00056-2 26364263 PMC4700047

[2] Molina JM Capitant C Spire B Pialoux G Cotte L Charreau I. On-Demand Preexposure Prophylaxis in Men at High Risk for HIV-1 Infection. N Engl J Med. 2015;373(23):2237-46. 10.1056/NEJMoa1506273 26624850

[3] Baeten JM Donnell D Ndase P Mugo NR Campbell JD Wangisi J Antiretroviral prophylaxis for HIV prevention in heterosexual men and women. N Engl J Med. 2012;367(5):399-410. 10.1056/NEJMoa1108524 22784037 PMC3770474

[4] World Health Organization (WHO). WHO expands recommendation on oral pre-exposure prophylaxis of HIV infection (PrEP). Copenhagen: WHO; 2015. Available from: https://www.who.int/hiv/pub/prep/policy-brief-prep-2015/en

[5] European Centre for Disease Prevention and Control (ECDC). Pre-exposure prophylaxis for HIV prevention in Europe and Central Asia. Monitoring implementation of the Dublin Declaration on partnership to fight HIV/AIDS in Europe and Central Asia - 2018/19 progress report. Stockholm: ECDC; 2019; Available from: https://www.ecdc.europa.eu/en/publications-data/ecdc-evidence-brief-pre-exposure-prophylaxis-hiv-prevention-europe-and-central

[6] Public Health England (PHE). PrEP impact trial. London: PHE. [Accessed: 25 May 2021]. Available from: www.prepimpacttrial.org.uk

[7] Joint United Nations Programme on HIV/AIDS (UNAIDS). HIV Prevention 2020 road map. Geneva: UNAIDS; 2017. Available from: https://www.unaids.org/en/resources/documents/2017/hiv-prevention-2020-road-map

[8] World Health Organization (WHO). WHO implementation tool for pre-exposure prophylaxis (PrEP) of HIV infection. Module 5: monitoring and evaluation. Geneva: WHO; 2018. Available from: https://apps.who.int/iris/bitstream/handle/10665/279834/WHO-CDS-HIV-18.10-eng.pdf?ua=1

[9] Hsu C Sandford BA. The Delphi technique: making sense of consensus. Practical Assessment, Research, and Evaluation. 2007;12(10).

[10] Tanton C Geary RS Clifton S Field N Heap KL Mapp F Sexual health clinic attendance and non-attendance in Britain: findings from the third National Survey of Sexual Attitudes and Lifestyles (Natsal-3). Sex Transm Infect. 2018;94(4):268-76. 10.1136/sextrans-2017-053193 28974552 PMC5969324

[11] O’Halloran C Owen G Croxford S Sims LB Gill ON Nutland W Current experiences of accessing and using HIV pre-exposure prophylaxis (PrEP) in the United Kingdom: a cross-sectional online survey, May to July 2019. Euro Surveill. 2019;24(48):1900693. 10.2807/1560-7917.ES.2019.24.48.1900693 31796157 PMC6891944

[12] O’Halloran C, Sun S, Nash S, Brown A, Croxford S, Connor N, et al. HIV in the United Kingdom: towards zero HIV transmissions by 2030. 2019 report. London: Public Health England; 2019. Available from: https://assets.publishing.service.gov.uk/government/uploads/system/uploads/attachment_data/file/858559/HIV_in_the_UK_2019_towards_zero_HIV_transmissions_by_2030.pdf

[13] Brady M, Rodger A, Asboe D, Cambiano V, Clutterbuck D, Desai M, et al. BHIVA/BASHH guidelines on the use of HIV pre-exposure prophylaxis (PrEP). Letchworth: British HIV Association; 2018. https://www.bhiva.org/file/5b729cd592060/2018-PrEP-Guidelines.pdf 10.1111/hiv.12718 30869189

